# Relative Roles of the Cellular and Humoral Responses in the *Drosophila* Host Defense against Three Gram-Positive Bacterial Infections

**DOI:** 10.1371/journal.pone.0014743

**Published:** 2011-03-03

**Authors:** Nadine T. Nehme, Jessica Quintin, Ju Hyun Cho, Janice Lee, Marie-Céline Lafarge, Christine Kocks, Dominique Ferrandon

**Affiliations:** 1 Equipe Fondation Recherche Médicale, UPR 9022 du Centre National de la Recherche Scientifique (CNRS), Institut de Biologie Moléculaire et Cellulaire du CNRS, Université de Strasbourg, Strasbourg, France; 2 Department of Pediatrics, Massachusetts General Hospital, Harvard Medical School, Boston, Massachusetts, United States of America; University of Cambridge, United Kingdom

## Abstract

**Background:**

Two NF-kappaB signaling pathways, *Toll* and *immune deficiency* (*imd*), are required for survival to bacterial infections in *Drosophila*. In response to septic injury, these pathways mediate rapid transcriptional activation of distinct sets of effector molecules, including antimicrobial peptides, which are important components of a humoral defense response. However, it is less clear to what extent macrophage-like hemocytes contribute to host defense.

**Methodology/Principal Findings:**

In order to dissect the relative importance of humoral and cellular defenses after septic injury with three different Gram-positive bacteria (*Micrococcus luteus, Enterococcus faecalis*, *Staphylococcus aureus*), we used latex bead pre-injection to ablate macrophage function in flies wildtype or mutant for various *Toll* and *imd* pathway components. We found that in all three infection models a compromised phagocytic system impaired fly survival – independently of concomitant *Toll* or *imd* pathway activation. Our data failed to confirm a role of the PGRP-SA and GNBP1 Pattern Recognition Receptors for phagocytosis of *S. aureus*. The *Drosophila* scavenger receptor Eater mediates the phagocytosis by hemocytes or S2 cells of *E. faecalis* and *S. aureus*, but not of *M. luteus.* In the case of *M. luteus* and *E. faecalis*, but not *S. aureus*, decreased survival due to defective phagocytosis could be compensated for by genetically enhancing the humoral immune response.

**Conclusions/Significance:**

Our results underscore the fundamental importance of both cellular and humoral mechanisms in *Drosophila* immunity and shed light on the balance between these two arms of host defense depending on the invading pathogen.

## Introduction

To combat infection, *Drosophila* relies on multiple defense reactions that can be grouped into three major arms: i) a systemic immune response in which the fat body (a functional equivalent of the mammalian liver) secretes into the hemolymph antimicrobial peptides (AMPs), ii) an enzymatic cascade leading to melanization at the site of wounding, and iii) a cellular response in which bacteria are phagocytosed by hemocytes (this study, [1]). The systemic immune response is triggered and regulated by two well studied NF-kappaB signaling pathways; the *Toll* and *imd* pathways [2]. The former is required to fight off some Gram-positive and fungal infections, while the latter plays a similar role in the host defense against Gram-negative bacteria. Mutations affecting molecular components of these pathways render flies generally more susceptible to either Gram-positive and fungal infections (*Toll*) or Gram-negative bacterial infections (*imd*).

The Pattern Recognition Receptors (PRRs) of the *imd* pathway, Peptidoglycan Recognition Protein-LC (PGRP-LC) and PGRP-LE, sense diaminopimelic acid-containing peptidoglycan (DAP-PGN) found for instance in Gram-negative bacteria [1], [2], [3]. These PRRs activate then the intracellular *imd* pathway through adapter proteins such as IMD and Kenny (KEY, also known as DmelIKKgamma), ultimately leading to the nuclear translocation of the Relish NF-kappaB transcription factor and the induction of multiple AMP genes such as *Cecropins*, *Attacins*, *Defensin*, *Drosocin,* and *Diptericin*.

The *Toll* pathway is activated upon binding of the Toll receptor to its mature ligand, Spätzle (SPZ), a cytokine of the Nerve Growth Factor family [1], [2], [3]. SPZ can be matured as the result of the activation of a proteolytic cascade initiated by a complex consisting of Gram Negative Protein Binding 1 (GNBP1) and PGRP-SA bound to the various Lysine-type peptidoglycans (Lys-PGN) found in many Gram-positive bacteria such as *Micrococcus luteus*, *Enterococcus faecalis,* and *Staphylococcus aureus*. Even though PGRP-SD does not bind strongly to Lys-PGN, it is required for sensing some Gram-positive bacterial infections by forming complexes with GNBP1 and PGRP-SA [4], [5]. In addition to binding Lys-PGN, PGRP-SA also binds to DAP-PGN with lower affinity [6], and, together with PGRP-SD, may mediate the weak activation of the *Toll* pathway by Gram-negative bacteria. Toll receptor activation leads to the nuclear uptake of the NF-kappaB transcription factors Dorsal and Dorsal-related Immune Factor (DIF), a process that requires the DmelMYD88 adapter. DIF appears to be the transcription factor that mediates Toll pathway activation during the immune response of adult flies, although Dorsal may play a weak, partially redundant role.

Biochemical and molecular biology approaches have led to the identification of multiple AMPs active, or thought to be active, on Gram-negative bacteria, namely Diptericin, Drosocin, Attacins, and Cecropins [7], [8], [9]. These AMP genes are regulated by the *imd* pathway, in keeping with the role of this pathway in the host defense against Gram-negative bacterial infections. In contrast, the AMP genes mainly controlled by the *Toll* pathway, *Drosomycin* and *Metchnikowin*, encode antifungal peptides, and not antibacterial peptides. The only *Drosophila* AMP active on Gram-positive bacteria identified to date, is Defensin [10]. Its expression, similar to those of *Attacins* and *Cecropins*, is decreased in *Toll* pathway mutants after an immune challenge with a mixture of *Escherichia coli* and *M. luteus* [7], [11], possibly reflecting a synergy between *Toll* and *imd* pathways in the case of mixed infections [12]. Because the *Toll* pathway is required in the host defense against Gram-positive bacteria, it is assumed that this partial control of *Defensin* by this pathway in the special case of mixed Gram-positive and -negative bacterial challenge is physiologically relevant, a notion reinforced by the finding that *Defensin* overexpression is sufficient to provide protection to *imd-Toll* pathway double mutant flies against several Gram-positive bacterial species [9].

In contrast to our knowledge of the systemic immune response, phagocytosis by macrophage-like cells remains less well characterized in *Drosophila*. Two studies underlined the importance of the cellular defense in larvae, which prevents microbes from colonizing the hemocoel and thereby ensures survival to imaginal stages [13], [14]. In adult flies, hemocytes are less abundant than in larvae and are mostly sessile [15]. Interestingly, blocking phagocyte function by the prior injection of latex beads in adult flies is not sufficient to confer susceptibility to *Escherichia coli* infections, unless performed in hypomorphic *imd* mutant flies [16]. This finding suggested that phagocytosis plays a minor role in the host defense against infections with Gram-negative bacteria that are sensitive to the humoral immune response. Several recent studies performed with more pathogenic bacteria suggest that the cellular arm of host defense plays a more important role in the response against some of these infections [17], [18], [19]. However, none of these recent studies directly addressed the relative contributions of the different arms of the immune response to host defense against bacterial infections *in vivo*. A variety of phagocytic receptors that can mediate the uptake of different classes of bacteria by hemocyte-like cell lines or primary macrophages have been identified in recent years, yet, their role in controlling infection *in vivo* remains unclear in most cases (Stuart and Ezekowitz, 2008).

In contrast, by using an intestinal model of infection with the Gram-negative entomopathogenic bacterium *Serratia marcescens*, we have established the essential role of phagocytosis and of the Eater phagocytic receptor in controlling the proliferation of bacteria that have crossed the intestinal barrier [20], [21]. Interestingly, the systemic immune response is not triggered by bacteria present in the hemocoel, leaving the cellular immune response as the only defense against bacterial proliferation in the insect body cavity [20], [21]. Eater, a novel phagocytic receptor of the scavenger family that displays broad specificity against Gram-negative and Gram-positive bacteria mediates predominantly the cellular response to ingested *Serratia* [20].

These findings raise the question whether phagocytosis may be important also in the *Drosophila* host defense against Gram-positive infections, which is poorly understood in terms of effector mechanisms. Indeed, while the *Toll* pathway is required in the host response against Gram-positive bacterial species, it remains unclear how it actually defends the host against microbial infections as Defensin is not necessary to mediate protection [22]. In addition, studies performed with *S. aureus* point out the existence of a PRR- dependent (PGRP-SA, PGRP-SD, GNBP1), but Toll-independent defense mechanism [5], [23].

Here, we show that *Drosophila* phagocytes play a central role in the host defense against three Gram-positive bacterial pathogens. The cellular immune response was mediated by the phagocytic receptor Eater for two of these bacterial species, but not a third, indicating some recognition specificity and providing an explanation for the existence of multiple phagocytosis receptors. Furthermore, we confirmed that Gram-positive bacteria sensing PRRs are required for controlling *S. aureus* independently of *Toll* pathway activation [5], [23] and provide evidence against an involvement of these PRRs in phagocytosis. Finally, we report that a defective cellular immune response to some Gram-positive bacterial species could be compensated by enhancing the humoral immune response.

## Results

### Phagocytosis plays a critical part in the host defense in adult *Drosophila* and acts independently of the antimicrobial peptide response

**Figure 1:**
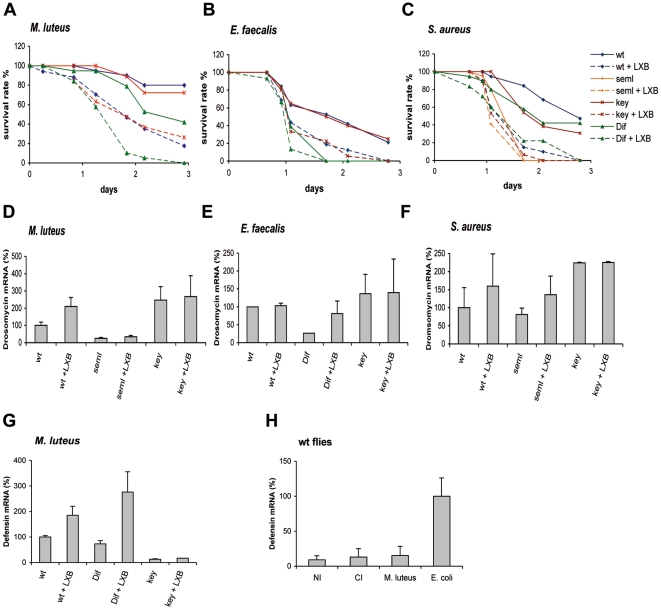
Phagocytosis in adult flies restricted Gram-positive bacterial infection independent of antimicrobial peptides induction. **A–C**. Flies were either preinjected with latex beads (LXB) or nontreated and then submitted to a septic injury with *M. luteus* (**A**), *E. faecalis* (**B**) and *S. aureus* (**C**). LXB pre-injected flies were significantly more susceptible to infection than noninjected wild type flies. (**A.** wt *vs*. wt + LXB : p<0.0001; *key vs. key* + LXB : p = 0.0003; *Dif vs. Dif* + LXB : p<0.0001. **B.** wt *vs*. wt + LXB : p = 0.02; *key vs. key* + LXB : p = 0.01; *Dif vs. Dif* + LXB : p = 0.08. **C.** wt *vs*. wt + LXB : p<0.0001; *key vs. key* + LXB : p = 0.0004; *seml vs. seml* + LXB : p = 0.02.) The survival rate expressed in percentage is shown. *wt*, wild-type controls. *Dif*, and *PGRP-SA^seml^* (*seml*) are mutants of the *Toll* pathway, whereas *key* (*kenny*) is a mutant of the *imd* pathway. Susceptibility of LXB-injected flies to *M. luteus,* although sometimes less pronounced (*e.g.,* Fig. 2, 3) was always statistically significant. **D-G.** LXB-preinjection did not impair *Drosomycin* or *Defensin* induction. Expression of the AMP gene was determined by real-time PCR. Results are expressed as a percentage of the induction observed in wt control flies. *Drosomycin* mRNA levels were monitored 24 hr after a challenge with *M. luteus* at 25 °C (D) and 48 hr after a challenge with *E. faecalis* or *S. aureus* at 20 °C (E and F). *Defensin* RNA levels were monitored 6 hr after a challenge with *M. luteus* at 25 °C (G). For *E. faecalis* or *S. aureus* the experiments were performed at a lower temperature because these bacteria are highly virulent, killing the flies rapidly. Error bars represent standard deviation (SD). **H.** Gram-positive bacteria did not induce *Defensin* expression. Expression of the AMP gene was determined by real-time PCR. Results are expressed as a percentage of the induction observed in wt control flies. *Defensin* RNA levels were monitored 6 hr after a clean injury (CI), a challenge with *M. luteus* or *E. coli* at 25 °C. Error bars represent SD.

In order to address the role of phagocytes in the *Drosophila* host defense to infection, we used a previously established assay to functionally ablate phagocytes by injecting latex beads (LXB) into the hemocoel of flies [16], [24]. Once engulfed by hemocytes, these beads block further phagocytosis, presumably because they cannot be degraded and metabolized. Flies injected with LXB 18 hours before an immune challenge were monitored for survival to infections after septic injury with three different Gram-positive bacteria : *M. luteus*, *E. faecalis,* and *S. aureus* (Fig. 1A). In all cases, LXB pre-injected flies were significantly more susceptible to infection than noninjected wild type flies (Fig. 1 A-C). To ensure that this increased sensitivity to infections did not result from our experimental procedures, we compared the survival of LXB-injected flies to phosphate-buffered-saline (PBS) injected flies after a *M. luteus* challenge and found that only the former succumbed (data not shown; see also below). Furthermore, LXB injection did not lead to significant lethality : LXB-injected, PBS-injected, and noninjected wild-type and *MyD88* flies survived equally well to a mock challenge (clean injury; data not shown). Finally, we checked that the increased sensitivity to infection when phagocytosis was blocked correlated with an increased bacterial titer. For instance, we found that 24 hours after the injection of about 100 *E. faecalis* cells, the bacterial titer per fly was 5 10^4^ on average in control wild-type flies whereas it was 35 fold higher in LXB-injected flies. Similarly, a 40-fold difference between control and LXB-injected flies was observed after a challenge with about 10 *S. aureus* cells. In contrast, we could not reliably measure a similar increase after a *M. luteus* challenge. These results suggest that functionally intact phagocytes constitute a critical component of the host defense against these Gram-positive bacteria.

To gain insight into the mechanism of this anti-bacterial response, we monitored in infected flies - in which the phagocytes had been functionally ablated by LXB pre-injection – the transcriptional induction of *Drosomycin* as a read-out of *Toll* pathway activation. LXB-preinjection did not impair *Drosomycin* induction in wild-type or *imd* pathway (*key)* mutant flies (Fig. 1 D-F). On the contrary, we noted a higher induction of the *Drosomycin* gene in LXB-injected flies in some experiments. Similarly, LXB-injection did not lead to a decreased induction of *Defensin*, a gene that appears to be controlled by the *imd* pathway as observed here in *key* mutants (Fig. 1G). It is noteworthy that septic injury with *M. luteus* does not induce *Defensin* expression above the level of a clean injury, which corresponds to only about 10% of the induction seen with *E. coli* (Fig. 1H). Together, these results suggest that phagocytes restrict bacterial infection independently of an AMP response, which is induced in the fat body.

This inference was further supported by the finding that LXB pre-injection also increased the susceptibility of mutants of the *Toll* and *imd* pathways (*Dif* and *key* respectively) to all three bacterial species (with the exception of *Dif* mutant flies that were killed by *E. faecalis* too rapidly) (Fig. 1A-C). Taken together, our results indicate that phagocytosis is an important immune defense mechanism in the adult fly and plays a critical and general role in restricting infections by these Gram-positive bacteria.

### The soluble pattern recognition receptors GNBP1 and PGRP-SA are unlikely to facilitate phagocytosis by functioning as major opsonins

**Figure 2:**
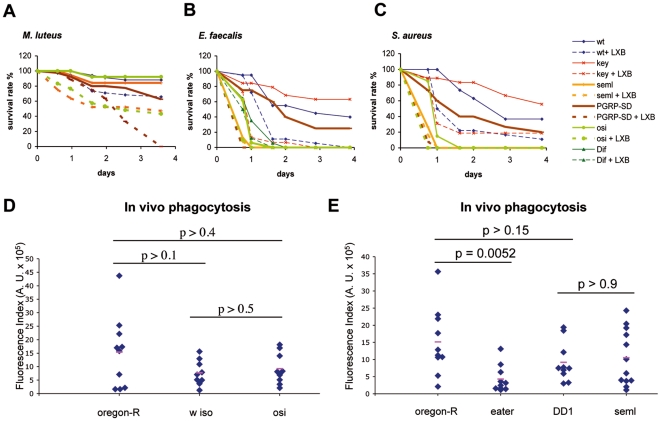
The soluble PRRs GNBP1, PGRP-SA, and PGRP-SD are unlikely to function as opsonins. A-C. Flies were either preinjected with latex beads (LXB) or nontreated and then submitted to a septic injury with *M. luteus* (A), *E. faecalis* (B) and *S. aureus* (C). LXB injection has a strong effect on the survival of *PGRP-SA^seml^* and *GNBP1^osi^* as well as *PGRP-SD^Δ3^* mutants after *M*. *luteus* infection (A). The results were less pronounced for *PGRP-SA^seml^* and *Dif* when we used *E. faecalis* (B) and *S. aureus* (C) as pathogens. (**A.** wt *vs*. wt + LXB : p = 0.01; *seml vs. seml* + LXB : p = 0.0005; *PGRP-SD vs. PGRP-SD* + LXB : p = 0.0004; *osi vs. osi* + LXB : p = 0.0001. **B.** wt *vs*. wt + LXB : p = 0.0005; *key vs. key* + LXB : p<0.0001; *seml vs. seml* + LXB : p = 0.26; *PGRP-SD vs. PGRP-SD* + LXB : p<0.0001; *osi vs. osi* + LXB : p = 0.001; *Dif vs. Dif* + LXB : p = 0.13. **C.** wt *vs*. wt + LXB : p = 0.004; *key vs. key* + LXB : p = 0.006; *seml vs. seml* + LXB : p = 0.49; *PGRP-SD vs. PGRP-SD* + LXB : p<0.0001; *osi vs. osi* + LXB : p<0.0001.) The survival rate expressed in percentage is shown. *PGRP-SD^Δ3^* (*PGRP-SD*); *GNBP1^osi^* (*osi*). **D, E.** Quantification of in vivo phagocytosis of Alexa-fluor labeled *S. aureus*. Each dot corresponds to the amount of fluorescence signal in the abdomen of one individual fly (a phagocytic index was derived by multiplying the area with the mean intensity of the fluorescence signal measured). Pair wise P-values are indicated by black bars. A horizontal red bar indicates the average phagocytic index for each group. No significant differences were observed between mutants and their corresponding wild-type controls (Oregon-R, w iso and DD1).

GNBP1, PGRP-SA, and PGRP-SD are Pattern Recognition Receptors (PRRs) that sense the presence of Gram-positive bacteria in the hemolymph and activate the *Toll* pathway via a proteolytic cascade. *GNBP1^osi^, PGRP-SD,* and *PGRP-SA^seml^* mutant flies succumb more rapidly to *S. aureus* infections than *Toll* pathway signaling mutants such as *Dif*, *MyD88*, and *spz* (Fig. 1C, [5], [23]), indicating that the GNBP1/PGRP-SA/PGRP-SD complex has Toll-independent functions in the host defense against some Gram-positive bacterial species. Indeed, it has been reported that *PGRP-SA* is required for the efficient phagocytosis of *S. aureus*, but not that of *E. coli*, suggesting that it might play a role in enhancing phagocytosis as an opsonin [25]. We reasoned, that if this were indeed the case, phagocyte ablation in mutant flies should not strongly increase susceptibility to infection. Therefore, we pre-injected mutant flies lacking PGRP-SA, GNBP1, or PGRP-SD expression with LXB and monitored their survival after septic injury with *M. luteus*, *S. aureus,* and *E. faecalis*. LXB-injected PRR mutant flies succumbed much more rapidly to a challenge with all three Gram-positive species than the respective nonLXB-injected mutants (except for *PGRP-SA^seml^* flies, which succumbed too rapidly to *E. faecalis* and to *S. aureus* in this series of experiments to observe an effect; Fig. 2A-C, but see below for another experiment in which the difference is observable). The finding that *GNBP1* and *PGRP-SD* mutant flies succumb more rapidly than wild-type flies to the three Gram-positive bacterial strains when phagocytosis is blocked suggests only a rather limited role, if any, for these PRRs in phagocytosis, at least with the bacterial pathogens tested.

To assess more directly a possible involvement of GNBP1 and PGRP-SA in phagocytosis, we tested the efficiency with which *GNBP1^osi^* and *PGRP-SA^seml^* hemocytes engulf fluorescently labeled *S. aureus* using a quantitative phagocytosis assay in living flies that allowed us to demonstrate *in vivo* the role of Eater in phagocytosis [20]. This assay may however not be sensitive enough to detect minor phenotypes. As shown in Fig. 2D and E, we could not detect any significant differences in bacterial uptake between mutants and their cognate wild-type controls. Hence, it is unlikely that a PGRP-SA/GNBP1 complex functions as a major opsonin for *S. aureus* in the *Drosophila* host defense.

### The phagocytic receptor Eater mediates host resistance to *E. faecalis* and *S. aureus*, but not to *M. luteus*

**Figure 3:**
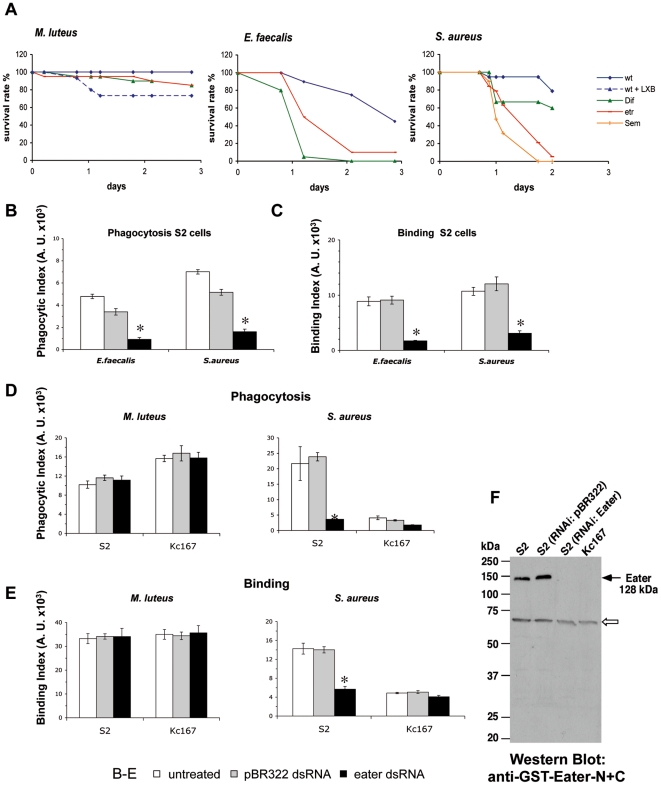
The phagocytic receptor Eater plays an important role in the *Drosophila* host defense against *E. faecalis* and *S. aureus* but not *M. luteus*. **A.** Flies were either preinjected with latex beads (LXB) or nontreated and then submitted to a septic injury with *M. luteus* (A), *E. faecalis* (B) and *S. aureus* (C). *Eater* mutant flies succumbed rapidly to a challenge with *S. aureus* and *E. faecalis* but not with *M. luteus*. (A. wt *vs*. wt + LXB : p = 0.0176; wt *vs. eater* : p = 0.0214. B. wt *vs. eater* : p = 0.0003. C. wt *vs. Dif* : p = 0.13; wt *vs. eater* : p<0.0001; wt vs. seml : p<0.0001). The survival rate expressed in percentage is shown. **B-E.** FACS analysis of phagocytosis and cell surface binding of heat-killed fluorescent bacteria to hemocyte-derived cell lines. To assess phagocytosis, extracellular fluorescence was quenched by trypan blue. The amount of phagocytosis (or cell surface binding) was quantified as percentage of cells phagocytosing (or binding) multiplied by mean fluorescence intensity. Error bars represent SD between four samples. * indicates : significantly different (p<0.01). **B, C.** RNAi knock down of Eater in S2 cells affects phagocytosis and binding of FITC-*E. faecalis* and *S. aureus.* **D, E.** RNAi knock down of Eater in S2 and Kc167 cells does not affect phagocytosis (D) and binding (E) of *M. luteus.* **F.** Eater protein is not detectable after RNAi knockdown in S2 cells and in Kc167 cells: Western Blot of cell extracts corresponding to 84 µg of protein separated on a 10% SDS-gel. A 128 kDa band corresponding to the Eater protein (black arrow) was present in S2 cells, whereas it was undetectable in S2 cells after RNAi knockdown of *eater*, or in untreated Kc167 cells. Control knockdown had no effect on *eater* expression. A nonspecific band at around 70 kDa (open arrow) served as an internal loading control.

To test whether the phagocytic receptor Eater plays a role in host defense to Gram-positive bacterial pathogens *in vivo*, we infected adult flies lacking the *eater* gene. Similarly to LXB-pre-injected flies, *eater* mutant flies succumbed rapidly to a challenge with *S. aureus* and *E. faecalis* (Fig. 3A). These data provide further evidence that phagocytosis is important to control these infections since Eater acts independently of the *Toll* and *imd* pathways as assessed by the normal induction of AMPs in *eater* mutants [20]. Similar results have been recently reported recently [26], [27].

However, unlike LXB-injected flies, *eater* flies were not, or only mildly affected by *M. luteus* infection (Fig. 3A), suggesting that Eater, despite its broad ligand specificity, is not important for phagocytosis of *M. luteus*. To further explore this question, we used a quantitative phagocytosis assay and RNA interference in cultured *Drosophila* S2 cells, a hemocytic cell line. In agreement with published results [20], *S. aureus* phagocytosis and binding to S2 cells was strongly dependent on Eater (Fig. 3B, C). Similarly, we found that *E. faecalis* was phagocytosed and bound to S2 cells in an Eater-dependent manner (Fig. 3B, C). In contrast to this, *eater* RNAi did not affect the uptake or binding to *M. luteus* into S2 cells (Fig. 3D, E). We also tested Kc167 cells, another *Drosophila* hemocyte cell line, in which Eater protein could not be detected (Fig. 3F). *M. luteus*, but not *S. aureus*, was efficiently bound and phagocytosed (in an *eater-*independent manner) in Kc167 cells (Fig. 3D, E). These data are consistent with the view that Eater is a phagocytic receptor with a broad ligand specificity and therefore generally important against a wide variety of bacterial pathogens. However, they also indicate that some bacteria (such as *M. luteus*), although not well recognized by Eater, are nevertheless efficiently phagocytosed, presumably through other phagocytic receptors expressed on hemocyte cell lines, and on primary hemocytes *in vivo*.

### Host resistance to some Gram-positive infections can be enhanced by strengthening the humoral response

**Figure 4:**
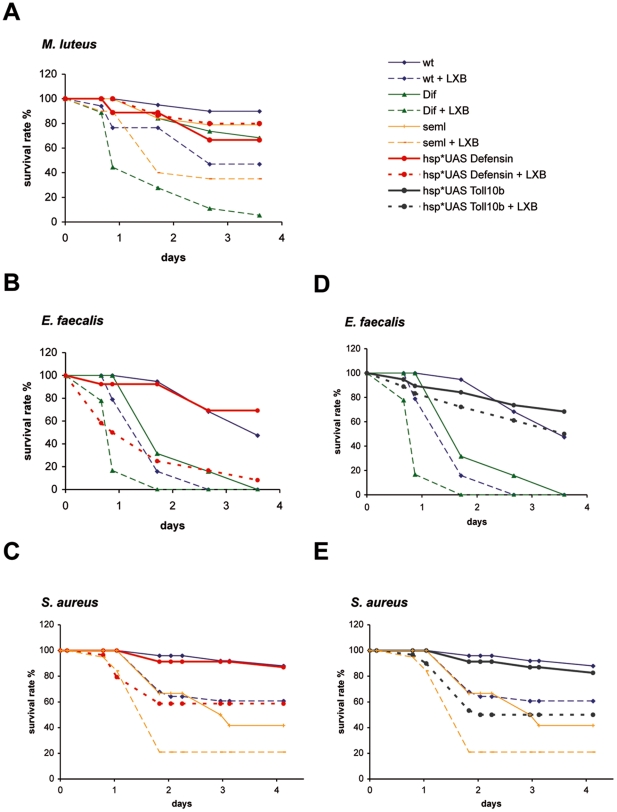
Overexpression of *Defensin* or Toll pathway can enhance host resistance to some Gram-positive bacteria. Flies were either preinjected with latex beads (LXB) or nontreated and then submitted to an immune challenge with *M. luteus* (A), *E. faecalis* (B and D) and *S. aureus* (C and E). LXB-injected flies in which *Defensin* was constitutively overexpressed (*UAS*-*Defensin*) using *hsp*-*GAL4* driver (*hsp*) were resistant to a *M. luteus* challenge (A). A protective effect was not observed for *E. faecalis* or *S. aureus* infections (B-C). LXB-injected flies in which Toll (UAS-*Toll^10b^*) was constitutively active were resistant to *E. faecalis*, but not to *S. aureus* (D-E). (**A.** wt *vs*. wt + LXB : p = 0.0014; *Dif vs. Dif* + LXB : p<0.0001; *seml vs. seml* + LXB : p = 0.002; hsp*UAS-*Defensin vs*. hsp*UAS-*Defensin* + LXB : p = 0.71; **wt + LXB** ***vs*** **.** hsp*UAS-Defensin **+ LXB : p = 0.03**. **B.** wt *vs*. wt + LXB : p<0.0001; *Dif vs. Dif* + LXB : p<0.0001; hsp*UAS-Defensin vs. hsp*UAS-Defensin + LXB : p<0.0001; **wt + LXB** vs. hsp*UAS-Defensin **+ LXB : p = 0.80**. **C.** wt *vs*. wt + LXB : p = 0.02; *seml vs. seml* + LXB : p = 0.09; hsp*UAS-Defensin vs. hsp*UAS-Defensin + LXB : p = 0.02; **wt + LXB** vs. hsp*UAS-Defensin **+ LXB : p = 0.55**. **D.** hsp*UAS- Toll^10b^ vs. hsp* UAS- Toll^10b^ + LXB : p = 0.25; **wt + LXB** vs. hsp* UAS-Toll^10B^ **+ LXB : p<0.0001**. **E.** hsp*UAS- Toll^10b^ vs. hsp* UAS- Toll^10b^ + LXB : p = 0.0015; **wt + LXB** vs. hsp* UAS-Toll^10B^ **+ LXB : p = 0.19**). The survival rate expressed in percentage is shown.

Phagocytosis is not required for the host defense against the weak Gram-negative pathogen *E. coli* but is required against both weak and potent Gram-positive pathogens ([16], this work). This situation may reflect a difference in the effectiveness of the humoral response mediated by the *imd* and *Toll* pathways respectively. We therefore asked whether we could experimentally compensate a phagocytosis defect by boosting the humoral response and first tested Defensin, which is the only AMP known so far with strong activity against Gram-positive bacteria [8], [9], [10]. As shown in Fig. 4A, flies in which Defensin was overexpressed using the UAS-Gal4 system prior to the immune challenge were resistant to a *M. luteus* challenge, even though phagocytosis had been inhibited by LXB injection (compare wt+LXB to hsp*UAS-Defensin+LXB). A similarly protective effect was not observed for *E. faecalis* or *S. aureus* infections (Fig. 4B, C). These data are partially in line with a previous study, which reported that the constitutive expression of *Defensin* protects *imd-spz* flies (which are fully deprived of a humoral immune response) from a challenge with *M. luteus* but protects against *S. aureus* only poorly [9].

Because the *Toll* pathway controls the expression of many genes in addition to AMPs [28], we asked whether the microbe-independent activation of the *Toll* pathway provided by a dominant allele of *Toll* (*UAS*-*Tl^10b^* transgene) could protect LXB-treated flies from an *E. faecalis* or a *S. aureus* challenge. As shown in Fig. 4D and E, the virulence of *E. faecalis*, but not that of *S. aureus*, was offset by the expression of a constitutively active form of Toll induced only at the adult stage. Indeed, LXB-treated hsp*UAS-*Tl^10b^* flies resisted an *E. faecalis* challenge better than wild-type or *Dif* flies in which phagocytosis had been inhibited by LXB injection. In contrast, LXB-treated flies expressing *Tl^10b^* were dying from *S. aureus* infection at the same rate as wild-type LXB*-*treated flies. Thus, an enhancement of the humoral immune response to fight off Gram-positive bacteria is an effective strategy against only some bacterial species.

## Discussion

In this work, we have directly investigated the relative contributions of the cellular and humoral facets of host defense against three species of Gram-positive bacteria that activate the *Toll* pathway. We find that phagocytosis plays an essential role against *M. luteus*, *E. faecalis*, and *S. aureus*. In contrast, as regards the humoral immune response in this study, *Toll* pathway mutants that affect signal transduction (mostly the intracellular part) are highly sensitive to *E. faecalis* and only weakly susceptible to *S. aureus*. In comparison, the *imd* pathway appears to play a leading role in the host defense against Gram-negative bacteria [1], [16]. The apparent prevalence of the *imd* pathway in the defense against Gram-negative bacteria is likely linked to its controls of multiple, fast evolving, AMPs induced in large quantities, making it difficult for pathogens to escape the antimicrobial activities [29]. In contrast, it is striking that in *Drosophila* only one AMP strongly active against Gram-positive bacteria, Defensin, has been identified to date by a biochemical approach [30], [31]. We report here that *Defensin* is not induced by a challenge with *M. luteus*, even though Defensin displays antibacterial activity against *M. luteus in vitro* and *in vivo* ([9,30, this work], this work). Thus, the Toll-dependent immune response does not appear to be adapted to Gram-positive bacteria as regards *Defensin* expression, even though *Drosophila* has evolved Lys-PGN sensors that activate the *Toll* pathway. *Defensin* expression may have been put under *imd* pathway control to fight Gram-positive bacterial infections in barrier epithelia in which the *imd*, and not the *Toll*, pathway appears to play a primary regulatory role [32], [33]. Alternatively, it may be an *imd-*dependent effector that fights off bacilli [9], which expose amidated DAP-type PGN on their cell wall.

*E. faecalis* is sensitive to the action of the *Toll* pathway and to the cellular immune response (this work, [22], [26], [34]). Moreover, both phenotypes appear to be additive, at least to some degree (Figs. 1, 3, 4). A defect in phagocytosis cannot be compensated by the overexpression of *Defensin* but can be rescued by the induced activation of the *Toll* pathway prior to infection. Because we are using a heat-shock promoter for the Gal4 line to drive UAS-*Tl^10b^* expression only at the adult stage, it is unlikely that the rescue we observed is due to indirect developmental effects. Note that *Defensin* is only mildly induced by *Toll* pathway constitutive activation [7]. Thus, it is likely that the activation of the *Toll* pathway leads to the expression of other effectors that are active on *E. faecalis* but that are not expressed at sufficient levels in the course of the response to an *E. faecalis* septic injury. The nature of these effectors remains to be established.

*S. aureus* is a potent pathogen in flies that is resistant to the action of the *Toll*-dependent immune response, a conclusion that is reinforced by the absence of protection provided by *Defensin* overexpression or *Toll* pathway constitutive activation when the cellular response is impaired (this work, [5], [23]). We report here that phagocytosis is able to control to some degree the speed of the infection and is thus a relevant host defense. Indeed, Avet-Rochex *et al.* have reported that flies in which phagocytosis is impaired either by the transgenic ectopic expression of the *Pseudomonas aeruginosa* RhoGAP ExoS in hemocytes or by mutations in the *rac2* gene are more susceptible to *S. aureus* infection [19], [35]. A susceptibility of PGRP-SC1a (*picky*) mutants to *S. aureus* infection has also been reported [25]. However, it is not fully clear whether the susceptibility of *picky* mutants to this pathogen is a consequence of impaired phagocytosis or defective *Toll* pathway activation that are reportedly both affected in this mutant [25], [36]. Finally, adult flies deprived of hemocytes are more sensitive to *S. aureus* infection [26].

What is the role of PGRP-SA and GNBP1 in the host defense against *S. aureus* since it is not *Toll* pathway activation? It has been proposed that PGRP-SA (and PGRP-SD) function as opsonins [25]. Our results (Fig. 2) do not support this suggestion. It is unlikely that these PRRs function to trigger the proteolytic cascades that activate melanization at the injury site because a sustained activation of the phenol oxidase activation cascade requires an intact intracellular *Toll* pathway [37], unlike the host defense against *S. aureus* in which the intracellular part of the *Toll* pathway is largely dispensable as observed in survival experiments (this work, [23]). Another hypothesis based on their specificity for cell wall components is that PGRP-SA and GNBP1, possibly with PGRP-SD, act directly as effector proteins, may-be by agglutinating bacteria as has been reported for other PRRs in insects [38], [39].

For two of the three Gram-positive bacteria tested here, *S. aureus* and *E. faecalis*, the phagocytic PRR Eater was found to mediate recognition and phagocytosis, *in vivo* in adult flies as well as *in vitro* in hemocyte-like S2 cells. These data strongly support the idea that Eater is important in host defense against a broad spectrum of bacteria, including various Gram-positive and Gram-negative bacteria [20]. Microbial recognition by Eater involves a direct interaction between its N-terminal four EGF-like repeats and microbial surfaces [20], and displays a multi-ligand specificity typical for scavenger receptors [40].

However, despite Eater's broad ligand specificity, phagocytosis of *M. luteus* was not dependent on Eater, neither *in vivo* nor *in vitro* in two different hemocyte-derived cell lines. Interestingly, the cell wall composition of the high G+C Gram-positive *M. luteus* (phylum *Actinobacteria*) differs from the low G+C Gram-positive *S. aureus* and *E. faecalis* (phylum *Firmicutes*). Peptidoglycan from *M. luteus* differs in the peptide bridges crosslinking the glycan strands [41], and *M. luteus* lacks the major cell wall components of most Gram-positive bacteria, teichoic acid and lipoteichoic acid, and instead uses two other classes of glycopolymers: teichuronic acid and lipomannan [42], [43]. Supporting the results of this study, we recently found that the N-terminus of Eater displayed direct binding to *S. aureus* and *E. faecalis* but not to *M. luteus* and interacted with polymeric peptidoglycan (or peptidoglycan-associated molecules) from *S. aureus* but not from *M. luteus* (Y.-S. A. Chung and C. Kocks, submitted). Our findings thus raise interesting questions to about the exact nature of the microbial components recognized by Eater, their presence or absence among Gram-positive surface structures and how this challenge of cell wall diversity is met by the phagocytic receptor repertoire in flies.

An array of diverse membrane-bound proteins has been implicated in phagocytosis in *Drosophila* in recent years (different scavenger receptors, other EGF-repeat receptors (Nimrods), the CD36 family member Peste, DSCAM, croquemort [44], [45], [46], [47], [48], [49]; for a recent review see Stuart & Ezekowitz [50]). It will be interesting to determine if any of these mediates recognition of *M. luteus* and *in vivo* host defense. Gram-positive bacteria are extremely diversified and abundant in soil and on decaying matter such as rotting fruit, the natural habitat of *D. melanogaster*. Since the *Toll* pathway does not appear to be as effective against Gram-positive bacteria as the *imd* pathway is against Gram-negative bacteria, Gram-positive bacteria may constitute a promising source of microorganisms to test the functions of putative phagocytosis receptors in *Drosophila* host defense.

In summary, our experiments reveal that phagocytosis plays a cardinal role in fighting off Gram-positive bacteria but that an impaired cellular immunity can be compensated for by strengthening the humoral immune response. This strategy functions only with bacteria that are susceptible to AMPs or other effectors of the *Toll* pathway. It is likely that a similar balance between these two facets of innate immunity exists for Gram-negative bacteria, except that it may be difficult for Gram-negative bacteria to resist the action of the *imd* pathway because it controls the expression of multiple AMPs. Pathogenic bacteria able to escape or resist the actions of the systemic humoral response may drive the evolution of phagocytic receptor loci by the interplay of host-pathogen interactions. Indeed, strong evidence for pathogen-driven positive selection in putative phagocytosis receptors has been observed in the 12 sequenced genomes of *Drosophila* species [29]. Based on our data, it is likely that a constitutive, stronger, or a more rapid activation of the *Toll* pathway could provide the fly with an added level of defense. This strategy has not been selected during evolution, possibly because *Drosophila* do not encounter in the wild at high enough a frequency bacteria that are resistant to the humoral immune response. Alternatively, the protection provided by enhanced *Toll* pathway activation may be metabolically too costly or even detrimental to the fitness of noninfected flies [51], [52], [53].

## Materials and Methods

### Microbial Strains

Gram-positive bacteria used in this study include *Micrococcus luteus* (CIP A270), *Enterococcus faecali*s and *Staphylococcus aureus* (kind gifts from H. Monteil, University Louis Pasteur, Strasbourg, France). Fluorescein isothiocyanate (FITC) and Alexa-Fluor 488**-**labeled *S. aureus* were purchased from Molecular Probes. For fluorescent labeling, bacteria were grown to early saturation, heat-killed at 70°C for one hour, washed, and labeled with FITC by standard procedures.

### Fly Strains

Stocks were raised on standard cornmeal-agar medium at 25 °C. *Dif^1^* and *key^1^* mutants, [11], [54], [55], *GNBP1^osi^*, *hsp-GAL4*, *PGRP-SA^seml^*, and *PGRP-SD* ^Δ3^ stocks have been described previously (all mutant alleles are genetic nulls) [23], [34], [56]. *eater* null flies (transheterozygous F1) were generated as described previously [20] from deficiency lines Df(3R)605 and Df(3R)TI-I (Bloomington stocks #823 and 1911)**.** Stocks used for overexpression analysis were generated using standard crosses. *hsp-Gal4* drivers were used to ubiquitously express the transgenes. For the survival assays, flies were incubated at 29 °C 48 h prior to the heat-shock. Heat shocks was performed as follows: 20 min at 37 °C, 30 min at 18 °C, 20 min at 37 °C. Flies were incubated at 29 °C overnight before performing the experiments.

### Induction of antimicrobial peptide response and infection assays

Antimicrobial peptide synthesis was analyzed by quantitative reverse transcription PCR as previously described [57]. In survival experiments, batches of 20–25 wild-type and mutant flies were challenged by septic injury using a needle previously dipped into a concentrated solution of bacteria. The vials were then put at 25 °C and the surviving flies counted as required. Flies were usually transferred to new vials every other day. Note that for *S. aureus* we usually used a solution with OD_600_ = 0.2. For phagocyte ablation experiments, surfactant-free red, 0.3 μm diameter CML latex beads (Interfacial Dynamics Corp.) were washed in PBS and used 4× concentrated in PBS (corresponding to 5 to 10% solids) and 69 nl were injected 18 to 24 hours before septic injury. Data are representative of at least three independent experiments.

### RNA interference analyses and phagocytosis assays

dsRNAs were synthesized, Flow cytometry-based phagocytosis and bacterial binding assays in cultured cells were performed as described [20], [58]. *In vivo* phagocytosis assays were performed as described previously [20].

### Western Blot

Cytoplasmic extracts were prepared with a non-denaturing cell lysis solution (CelLytic M; Sigma) in the presence of protease inhibitor cocktail (Roche Applied Sciences). Proteins were separated by SDS-PAGE and transferred to PVDF membrane, and western blots developed using chemiluminescence. Anti-*eater* antiserum: N-terminal and C-terminal Eater domains corresponding to amino acids 19 to 58 and 1179 to 1206 were fused separately to glutathione-*S*-transferase (GST), overexpressed in *E. coli*, purified, mixed together and used to generate rabbit antiserum (anti-GST-Eater-N+C). Antibodies were purified using Protein A. Control Western Blots with truncated Eater molecules (purified soluble N-terminal fragment 1-199 or transfected C-terminal fragment 1024-1206) confirmed recognition of the mature N-terminus of Eater, as well as the C-terminal tail (data not shown).

### Statistical analysis

Statistical significance of survival experiment was calculated using the product limit method of Kaplan and Meier using the logrank test (GraphPad PRISM 4 software). Statistical significance of *in vivo* phagocytosis assay was assessed by calculating two-tailed p-values by a non-parametric rank sum test (Mann-Whitney U-test). p<0.05 is significant.
